# Incidence, electrophysiological characteristics, and long‐term follow‐up of perimitral atrial flutter in patients with previously confirmed mitral isthmus block

**DOI:** 10.1002/joa3.12545

**Published:** 2021-05-12

**Authors:** Panagiotis Ioannidis, Evangelia Christoforatou, Theodoros Zografos, Panagiotis Charalambopoulos, Konstantinos Kouvelas, Georgios Christoulas, Periklis Syros, Georgios Tsitsinakis, Theodora Kappou, Andreas Tsoumeleas, Sotirios Floros, Dimitrios Tagoulis, Ioannis Ntarladimas, Ioannis Tagoulis, Dimitrios Avzotis, Antonis S. Manolis, Charalambos Vassilopoulos

**Affiliations:** ^1^ Heart Rhythm Center Athens Bioclinic Athens Greece; ^2^ First Cardiology Department Red Cross Hospital Athens Greece; ^3^ First Department of Cardiology Athens University School of Medicine Athens Greece

**Keywords:** atrial fibrillation, atrial tachycardias, catheter ablation, linear lesions, mitral isthmus, perimitral atrial flutter, pseudo‐block

## Abstract

**Introduction:**

After mitral isthmus (ΜΙ) catheter ablation, perimitral atrial flutter (PMF) circuits can be maintained due to the preservation of residual myocardial connections, even if conventional pacing criteria for complete MI block are apparently met (MI pseudo‐block). We aimed to study the incidence, the electrophysiological characteristics, and the long‐term outcome of these patients.

**Methods:**

Seventy‐two consecutive patients (mean age 62.4 ± 10.2, 62.5% male) underwent MI ablation, either as part of an atrial fibrillation (AF) ablation strategy (n = 35), or to treat clinical reentrant atrial tachycardia (AT) (n = 32), or to treat AT that occurred during ablation for AF (n = 5). Ιn all patients, the electrophysiological characteristics of PMF circuits were studied by high‐density mapping.

**Results:**

Mitral isthmus block was successfully achieved in 69/72 patients (95.6%). Five patients developed PMF after confirming MI block. In these patients, high‐density mapping during the PMF showed a breakthrough in MI with extremely low impulse conduction velocity (CV). In contrast, in usual PMF circuits that occurred after AF ablation, the lowest CV of the reentrant circuit was of significantly higher value (0.07 ± 0.02 m/s vs 0.25 ± 0.07 m/s, respectively; *P* < .001). Patients presented with clinical AT had better prognosis in maintaining sinus rhythm after MI ablation compared with patients presented with AF.

**Conclusion:**

Perimitral atrial flutter with MI pseudo‐block may be present after MI ablation and has specific electrophysiological features characterized by remarkably slow CV in the MI. Thus, even after MI block is achieved, a more detailed mapping in the boundaries of the ablation line or reinduction attempts may be needed to exclude residual conduction.

## Graphical abstract

This is a retrospective clinical study comprising 72 patients who underwent mitral isthmus (MI) ablation either as part of an atrial fibrillation ablation strategy, or to treat reentrant AT. The MI block was acutely achieved in 69/72 patients. However, five patients, despite showing MI block based on conventional pacing maneuvers, developed perimitral atrial flutter through a gap in previous ablation lesions with extremely low impulse conduction velocity as shown by high density mapping.

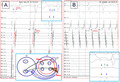

**Abbreviations**
AF: atrial fibrillation
AT: atrial tachycardia
CL: cycle length
CS: coronary sinus
CTI: cavo‐tricuspid isthmus
CV: conduction velocity
FTI: force‐time integral
ICE: intracardiac echocardiography
LA: left atrium
LAA: left atrial appendage
LAO: left anterior oblique
LAT: local activation time
ΜΙ: mitral isthmus
PMF: perimitral atrial flutter
PV: pulmonary vein
PVI: pulmonary vein isolation
SR: sinus rhythm

## 1 INTRODUCTION

The mitral isthmus (MI) is the anatomical area between the ostium of the left inferior pulmonary vein (PV) and the posterolateral part of the mitral valve annulus. MI ablation is an established strategy in the treatment of perimitral atrial flutter (PMF)^1^, ^2^ as well as an adjunct to pulmonary vein isolation (PVI) in the treatment of non‐paroxysmal atrial fibrillation (AF).^3^, ^4^

The usefulness of the MI ablation and generally of linear lesions is questionable as an initial strategy in persistent AF ablation.^5^, ^6^ However, it is one of the possible options in cases where PVI alone is not considered sufficient, such as in redo cases with well‐maintained PVI,^7^ long‐lasting persistent AF,^8^, ^9^ or diseased myocardium with low‐voltage electrical activity.^10^ On the other hand, MI ablation is an effective therapy for PMF; however, these reentrant tachycardias can also be treated by ablating critical isthmuses with slow conduction zones.^11^, ^12^, ^13^

If a linear lesion is attempted in the ΜΙ, the creation of complete and bidirectional block is very important. Failure to achieve bidirectional block can be proarrhythmic, and therefore, if it cannot be achieved, it should not even be attempted.^14^, ^15^, ^16^ The anatomical complexity of the MI hinders the creation of transmural lesions.^17^ This can lead to the persistence of conduction gaps that allow the maintenance of PMF despite the apparent evidence of complete block. This is a condition often referred to as MI pseudo‐block.^18^, ^19^, ^20^, ^21^

In this prospective study, we investigated clinical and electrophysiological characteristics of patients who had undergone MI ablation, focusing in particular on the group of patients who continued to have PMF, while MI block had been previously demonstrated by evidentiary pacing maneuvers. At the same time, we tried to peruse standard PMF circuits by measuring conduction velocities (CVs), in order to understand the specific electrophysiological properties of these reentrant tachycardias and to improve our knowledge in selecting the most appropriate PMF ablation strategy.

## 2 METHODS

### 2.1 Patient population

The study population comprised consecutive patients who underwent for the first time MI catheter ablation, from January 2018 to January 2020, either as part of a specific AF ablation strategy, or to treat clinical reentrant atrial tachycardia (AT), or to treat AT that occurred during ablation for AF. All patients underwent baseline evaluation with clinical history, physical examination, electrocardiogram, and transthoracic echocardiogram. The definitions and terminology regarding AF in this article are in agreement with the latest HRS/EHRA/ECAS/APHRS/SOLAECE Expert Consensus Statement on Catheter and Surgical Ablation of AF.^22^ All patients provided written informed consent. The study protocol was approved by the hospital's institutional review board. The study complied with the Declaration of Helsinki.

### 2.2 Electrophysiological study

All antiarrhythmic medications except for amiodarone were discontinued at least five half‐lives before ablation. All patients were on oral anticoagulant therapy which was not interrupted for the procedure. For patients taking vitamin K antagonists, an international normalized ratio between 2.0 and 3.0 was a prerequisite for the preoperative period as well as for the day of the procedure. For patients taking direct oral anticoagulants with twice‐daily dosing, the morning dose was also given in the majority of the patients; however, in a small minority, a single dose was held on the day of the procedure. Transesophageal echocardiography was performed in all patients just before the start of the procedure to exclude left atrial (LA) thrombi. The ablation procedure was performed under general anesthesia.

Twelve‐lead ECG and intracardiac electrograms were continuously monitored and stored on a computer‐based digital amplifier/recorder system (EP Workmate Recording System; Abbott). Intracardiac electrograms were filtered from 30 to 500 Hz. The EnSite Precision three‐dimensional system (Abbott) was used for mapping of the left and the right atrium if appropriate.

Barium ingestion, before the general anesthesia, was used for real‐time fluoroscopic imaging of the esophagus. A 100 IU/kg dose of heparin was administered immediately after the insertion of the femoral sheaths. Subsequent boluses of heparin were administered every 1 hour targeting an activated clotting time >300 seconds.

The following diagnostic catheters were introduced via the femoral veins: (1) a quadripolar catheter was positioned in the His bundle area; (2) a deflectable decapolar 6F catheter, electrode spacing 2‐5‐2 mm (Inquiry L; Abbott) was positioned in the coronary sinus (CS), with the distal electrode reaching the 4 o'clock position along the mitral annulus in the 30° left anterior oblique (LAO) radiographic projection.

A transseptal puncture was carried out with the use of an intracardiac echocardiography catheter (ViewFlex Xtra; Abbott) connected with a compatible ultrasound console (Philips CX50). A 20‐pole, variable radius circumferential mapping catheter (Reflexion Spiral; Abbott) was introduced with the help of a long transseptal sheath (Preface, Biosense Webster, or LAMP 45°; Abbott) to guide PVI, geometry creation, and high‐density activation and voltage mapping.

Α contact force ablation catheter (TactiCath Sensor Enabled; Abbott) was inserted into the left atrium (LA) through a steerable transseptal sheath (Agilis 71 cm; Abbott). An anatomical three‐dimensional left atrial (LA) model was systematically created by first inserting the ablation catheter in all PVs and the LA appendage (LAA), and then, by precisely delineating the body of the LA with the circumferential mapping catheter.

For the electrophysiological evaluation of Ats, local activation time (LAT) and propagation maps, as well as conventional entrainment mapping, were used to clarify the AT mechanism. A focal AT was defined when the activation map was indicative of centrifugal activation from a distinct source, while a macro‐reentrant tachycardia was diagnosed with 3D propagation maps when a discrete impulse rotation was evident corresponding to the entire cycle length (CL) of the tachycardia. Conventional entrainment mapping contributed to the diagnosis or was omitted if the propagation map was definitively diagnostic.

### 2.3 Catheter ablation

At the initial AF ablation procedure, a wide antrum PVI was attempted. After placing the 20‐polar circumferential PV mapping catheter in each PV, a point‐by‐point circumferential lesion encompassing the ipsilateral PVs was created aiming at complete electrical PV disconnection. RF energy was applied with an upper power of 30 W, a temperature limit of 40C° and saline irrigation flow 17 ml/min during RF delivery. A force of 10‐30 g and a force‐time integral (FTI) > 400 gs were targeted in every ablation lesion. If the ablation catheter was in close proximity to the esophagus, which was fluoroscopically visible with barium, the power was reduced to 25 W. In cases of non‐paroxysmal AF, after PVI, additional ablation of complex fractionated atrial electrograms or linear lesions were attempted at the discretion of the operators.

At the redo procedures, the PV antra were meticulously re‐mapped for conduction recurrence, and if observed, additional lesions were administered, using the above ablation settings, to achieve PVI. Previously administered linear lesions were also checked for bidirectional block, and in the case of residual conduction, further lesions were delivered. If the recurrent clinical arrhythmia was persistent AF, beyond the re‐isolation of the PVs, box lesion in the LA posterior wall, MI ablation, and possibly cavo‐tricuspid isthmus (CTI) ablation were performed. If the clinical arrhythmia was AT, the mechanism of the tachycardia was targeted in an individualized manner. For focal tachycardias, the area of origin was ablated, while for macro‐reentrant tachycardias, complete linear lesions were performed in anatomical isthmuses (MI, LA roof, CTI). A reinduction protocol with atrial extra and high‐rate stimulation was applied if any ΑΤ had occurred clinically or during the procedure.

### 2.4 Mitral isthmus ablation

The MI ablation was preferably performed in sinus rhythm (SR). Special emphasis was given on the accuracy of LA geometry in the MI area, and if there were obvious deficiencies, points were re‐collected to correct the 3D model. The MI line started at the 4 o'clock position of the mitral annulus (LAO view) and ended at the lower portion of the circular isolation line of the left PVs. With the ablation catheter supported by the steerable sheath, a contact force of 15‐35 g was applied in every ablation point. If the achieved pressure was less than 15 g, the ablation lesion was not attempted to avoid edema which could potentially hinder the formation of a transmural lesion. The radiofrequency (RF) application started from the ventricular aspect of the mitral annulus at the point where the atrial potential was apparent. The RF energy was delivered with an FTI target of 400‐1000 gs at each point, until the elimination of local electrogram or its reduction by 90%. After the completion of MI linear ablation, point by point mapping was performed along the MI line, and reinforcement ablation was given to any site with residual high‐frequency potentials or electrograms of an amplitude >0.05 mV. After elimination of the atrial potentials from the endocardial site, and if complete block had not been achieved, the ablation catheter was inserted through the CS reaching from the epicardium the level of the endocardial MI line. At this point and if discrete atrial potential was recorded, RF energy was delivered for maximum 15 seconds, with contact force of less than 30 g, power of 25 W, a temperature limit of 40°C and saline irrigation flow 17‐30 mL/min. The energy delivery at the epicardial site was continued until complete block attainment or elimination of the atrial potentials.

**FIGURE 1:**
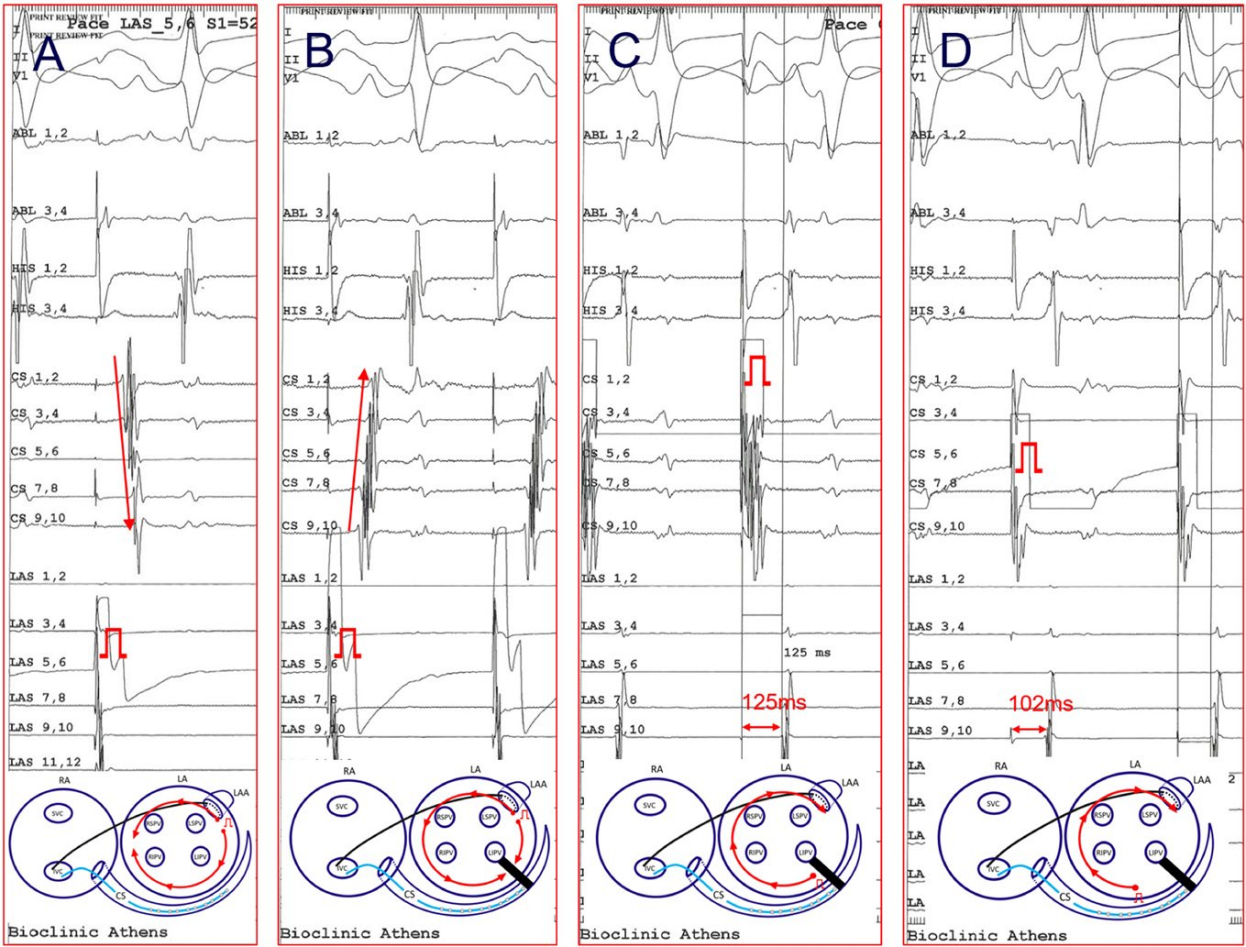
The pacing maneuvers performed in all patients to confirm mitral isthmus (MI) bidirectional block. Pacing superiorly to MI line before (A) and after (B) ablation to prove conduction block in the clockwise direction. Differential pacing from coronary sinus to prove conduction block in counterclockwise direction (C, D)

The complete and bidirectional MI block was verified by pacing maneuvers. For the implementation and interpretation of the pacing maneuvers, special care was taken to ensure that the pacing and recording catheters were as close as possible to the MI line. By pacing at CL of 500‐600 ms from the LAA or a point superiorly and close to the line, we sought complete and distinct reversal of the activation sequence in the CS. Additionally, differential pacing from the distal and an adjacent CS bipole would confirm conduction block in the counterclockwise direction (Figure 1). When the MI line was made during PMF, the termination of the tachycardia was further confirmatory of the PMF diagnosis, but the complete MI block had to be proven in SR by the pacing maneuvers described above. In all patients, the endocardial MI line length (longitudinal distance from mitral annulus to left inferior PV), and MI line width (maximum transverse distance from the anterior to posterior border of the ablated area) were measured, on the geometry created by the 3D system. The endocardial and epicardial RF ablation time were also measured.

### 2.5 Conduction velocity measurement

After the construction of the LAT map and the confirmation of the PMF diagnosis, a careful observation of the propagation map was carried out in order to identify areas with apparent delay throughout the whole wavefront course in a complete tachycardia circle. In these areas, a manual point correction was carried out at each received point, by locating the first deflection of the local electrogram. The timing scale was set either by spreading the color palette over the entire CL of the tachycardia or by defining color changing steps of 20 ms In the derived map, the shortest transverse distance in the direction of the wavefront propagation of an isochronal step was measured. For CV measurement, this distance was divided by the time interval of isochronal step and expressed in m/s. From the analyzed areas, the sites with the lowest CV were pointed out. In the inspection of the propagation map, channels of conduction with a width ≤1 cm were sought, and the impulse CV was also measured in these channels. Eligible for this analysis were maps containing more than 2000 points with sufficient dispersion throughout the circuit course.

### 2.6 Follow‐up

After ablation, antiarrhythmic drugs could be administered at the discretion of the electrophysiologists for a period of 2‐3 months. Anticoagulants were continued for the first 2‐3 months and stopped when the CHA2DS2VASC score was <1. All patients underwent follow‐up at 1, 3, 6, and 12 months following the ablation procedure and yearly thereafter. During the entire post‐procedural period, the patients were also followed by their referring physicians. Subsequent information was obtained by phone and/or e‐mail. Follow‐up visits included clinical examination, an ECG recording, and 24‐hour Holter monitoring. Tachycardia recurrence was defined as any atrial tachyarrhythmia (AT or AF) episode lasting ≥30 seconds documented in an ECG or Holter recording or during interrogation of an implantable cardiac rhythm device. A 3‐month time interval after MI ablation was defined as blanking period.

### 2.7 Statistical analysis

Continuous variables are summarized as mean ± SD or as median (interquartile range) according to data normality and were compared using Student's *t* test or Mann‐Whitney rank‐sum test, as appropriate. Categorical data are summarized as frequencies and percentages and were compared using Pearson chi‐squared test and Fisher's exact test. Between‐group differences were assessed using one‐way ANOVA or the Kruskal‐Wallis test, according to data normality. Rates for freedom from arrhythmia recurrence were determined using Kaplan‐Meier analysis and were compared by the log‐rank test across groups. Data analyses were performed using IBM SPSS Statistics version 25 (Armonk, NY, USA). A *p* value < 0.05 was considered statistically significant.

## 3 RESULTS

### 3.1 Patient characteristics and interventional outcome

Seventy‐two consecutive patients underwent MI catheter ablation either as part of a specific AF ablation strategy (n = 35), or to treat clinical reentrant AT (n = 32), or to treat AT occurred during ablation for AF (n = 5). Patient baseline characteristics are shown in Table 1. According to the predefined pacing maneuvers (Figure 1), the MI block was successfully achieved in 69 out of 72 (95.8%) patients (in 68 after the first procedure and in one patient after the second procedure). Of the four patients in whom complete MI block was unsuccessful during the first procedure, one had hypertrophic cardiomyopathy (MI block was achieved during the second ablation procedure); one suffered from cardiac amyloidosis (diagnosed later), while in the other two, the access to CS was not achieved due to a CRT‐D left ventricular lead, and a particular anatomical configuration, respectively. Of the three patients whose MI block was not finally achieved, only one had PMF treated with an anterior linear lesion at the same procedure, and since then the patient remains in SR. The mean MI ablation time of was 10.73 ± 6.16 minutes which was consisted of 10.12 ± 5.87 minutes for endocardial ablation and 1 ± 0.54 minutes for epicardial ablation through the CS.

**TABLE 1:** Patient baseline characteristics

	Total (n = 72)	Clinical arrhythmia AF (n = 40)^a^	Clinical arrhythmia AT (n = 32)	*P* value
Age (y)	62.4 ± 10.1	61.2 ± 11.4	63.9 ± 8.3	.2656
Male gender, n (%)	48 (66.7%)	27 (67.5%)	21 (65.6%)	.9350
Arterial hypertension, n (%)	44 (61.1%)	25 (62.5%)	19 (59.4%)	.9802
Diabetes mellitus, n (%)	6 (8.3%)	3 (7.5%)	3 (9.4%)	.8894
Coronary artery disease, n (%)	8 (11.1%)	2 (5%)	6 (18.8%)	.1408
Pacemaker or ICD, n (%)	3 (4.2%)	1 (2.5%)	3 (9.4%)	.4522
Left ventricular ejection fraction < 45%, n (%)	8 (11.1%)	6 (15%)	2 (6.3%)	.4300
Left atrial diameter (mm)	43.7 ± 2.7	43.7 ± 2.8	43.8 ± 2.6	.8770
Left ventricular ejection fraction (%)	52 ± 5.5	51.5 ± 4.7	52.7 ± 6.5	.3667
CHA_2_DS_2_‐VASc score	1.86 ± 1.28	1.8 ± 1.29	1.94 ± 1.29	.6487
Previous AF ablation, n (%)				
Total patients, n (%)	37 (51.4%)	9 (22.5%)	28 (87.5%)	<.0001
Patients with 1 previous procedure, n (%)	33 (45.8%)	8 (20%)	25 (78.1%)	<.0001
Patients with 2 previous procedures, n (%)	4 (5.5%)	1 (2.5%)	3 (9.4%)	.4522
Type of AF at the time of MI ablation				
Persistent AF, n (%)	21 (29.2%)	21 (52.5%)	0	—
Long‐standing persistent AF, n (%)	16 (22.2%)	16 (40%)	0	—
Paroxysmal AF, n (%)	3 (4.2%)	3 (7.5%)	0	—

Abbreviations: AF, atrial fibrillation; AT, atrial tachycardia.

^a^ This category includes also the five patients with AT occurred during ablation but had AF as a clinical arrhythmia.

### 3.2 Incidence of PFM with MI pseudo‐block

**FIGURE 2:**
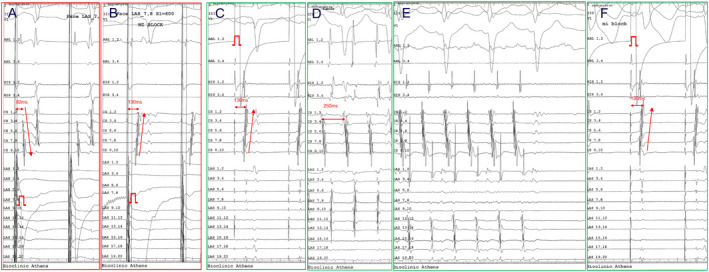
Pacing maneuvers indicative of mitral isthmus (MI) block and perimitral atrial flutter (PMF). (A) Pacing from left atrial appendage (LAA) with MI conduction before MI ablation in the first procedure. (B) Pacing from LAA with complete reversal in coronary sinus sequence after MI ablation in the first procedure. (C) Pacing superiorly of the MI line at the beginning of the second procedure before any ablation. (D) Clockwise PMF with cycle length = 250 ms (E) Termination of the arrhythmia by ablating the gap in MI (Video 3). (F) Reconfirmation of MI block

**FIGURE 3:**
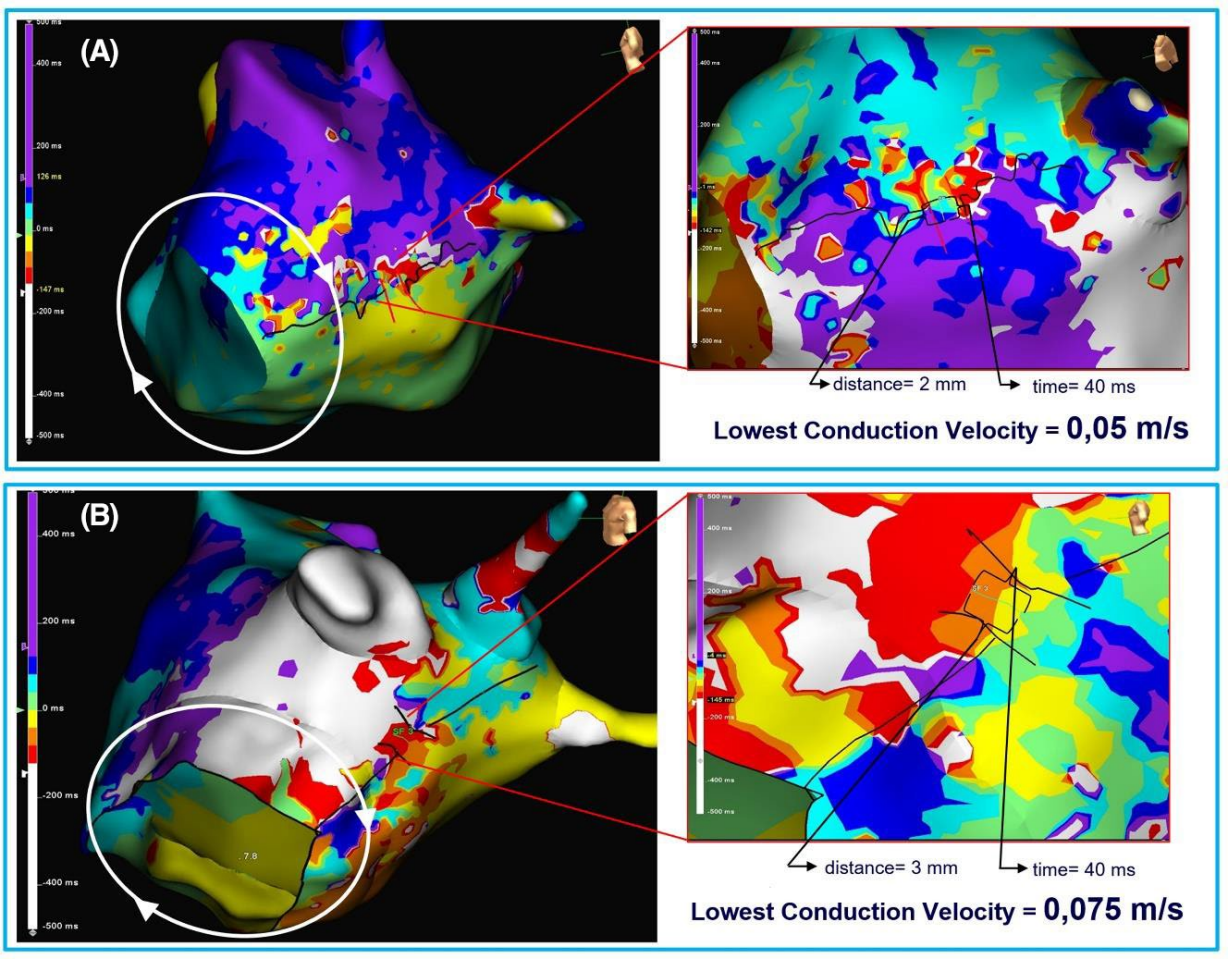
Isochronal maps in two cases (A, B) of clockwise perimitral atrial flutter circuits and mitral isthmus (MI) pseudo‐block showing the impulse breakthrough from a narrow channel in the MI with very low conduction velocity (Videos 1 and 4, respectively)

Despite the confirmation of MI block with the aforementioned pacing maneuvers, five patients presented with PMF. In three of these patients, PMF was emerged during the MI ablation procedure, after reinduction attempts with burst pacing, while two patients had recurrence at 3 and 6 months after the MI ablation procedure, and the diagnosis of PMF was made during the redo procedure, while pacing maneuvers were false positive for MI block before any ablation attempt (Figure 2). In all five patients, the detailed mapping showed a MI isthmus breakthrough with slow conduction along a narrow channel (Figure 3; Video a4). The endocardial ablation at this area restored the completeness of the MI line in all these patients. The complete and bidirectional block of the MI was re‐evaluated by pacing maneuvers and careful endocardial and epicardial mapping in the boundaries of the ablation line. In addition, after programmed atrial stimulation, PMF was no longer inducible.

### 3.3 PMF electrophysiological characteristics

**FIGURE 4:**
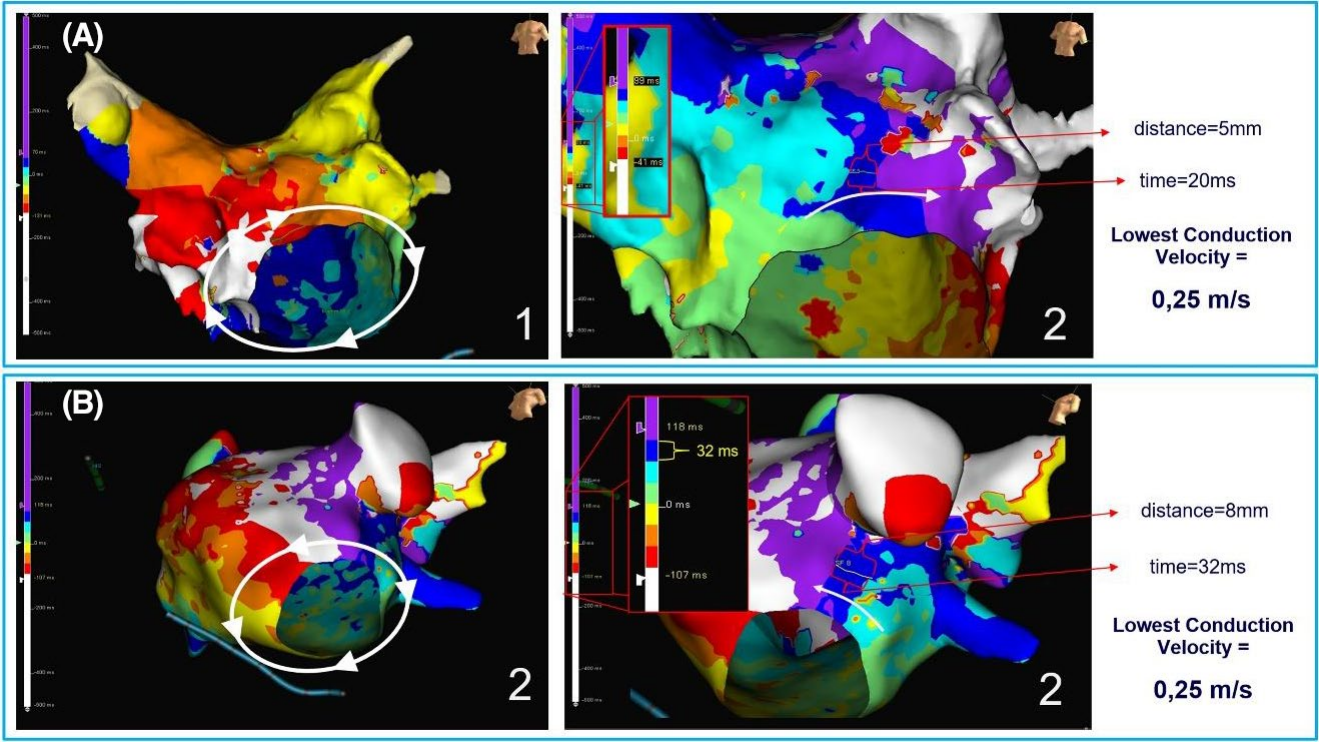
Isochronal maps of clockwise (Α1) and counterclockwise (Β1) perimitral atrial flutter with the corresponding measurement of the lowest conduction velocity (CV) in each circuit (A2, B2) (Videos 5 and 6)

**FIGURE 5:**
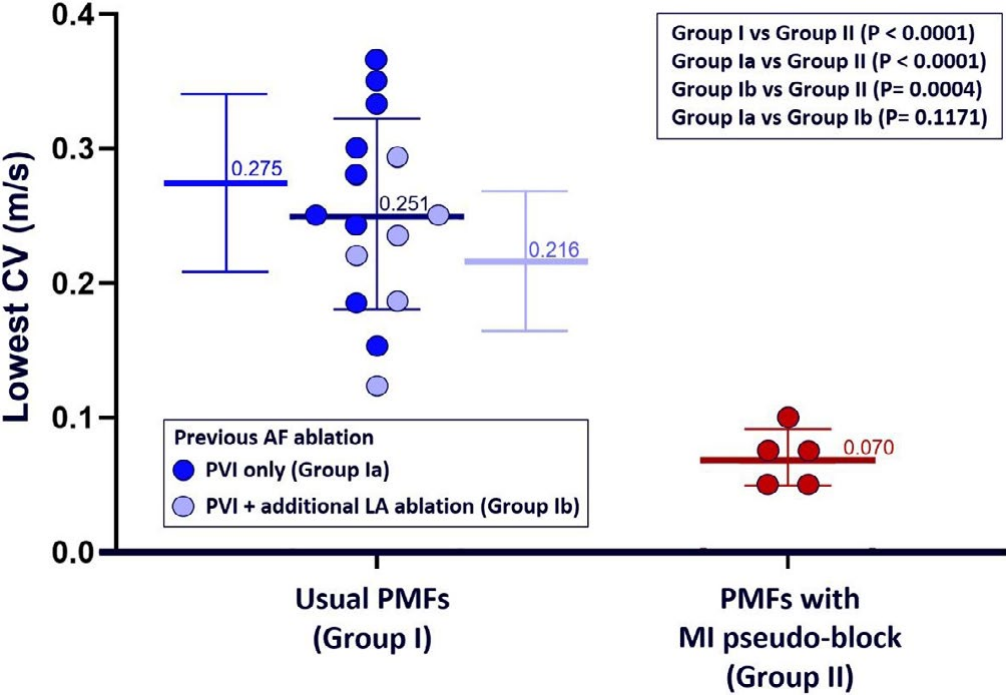
Comparison of the lowest CVs in usual perimitral atrial flutter (PMF) circuits and in PMF circuits after confirmed mitral isthmus block

During the procedures, 32 PMF (25 clockwise and seven counterclockwise) were detected in 28 patients (24 with AT and four with AF as the clinical arrhythmia). In 20 of the 32 PMF circuits (16 clockwise and four counterclockwise), it was possible to construct a high‐density map and measure the CVs as described earlier (Figure 4; Videos 5 and 6). The mean CL of PMFs with pseudo‐block (all clockwise) did not differ significantly from the mean CL of usual PMFs (254 ± 10 ms vs. 248 ± 13 ms, respectively; *P* = .340). However, the lowest CV in PMFs with pseudo‐block was of significantly lower value in comparison with the lowest CV in other thoroughly mapped PMF circuits (0.07 ± 0.02 m/s vs. 0.25 ± 0.07 m/s, respectively; *P* < .001). The same was true for patients with PMF who had undergone additional LA ablation beyond PVI (Figure 5). Conduction channels with width ≤1 cm were found in three patients with PMF without MI pseudo‐block. In these channels, the mean CV was 0.34 ± 0.08 m/s (range: 0.25‐0.4 m/s) (Figure S1; Video 7). In patients with PMF and ΜΙ pseudo‐block, the comparison of the ΜΙ length as well as the MI width of the ablation area showed no difference compared to patients with successful MI ablation. However, the width of the MI ablation area was significantly longer in patients with unsuccessful MI ablation (Table 2).

**TABLE 2:** Patient characteristics and outcome according to the interventional outcome of MI ablation

	Total (n = 72)	Group A Successful^a^ MI ablation without PMF recurrence (n = 62)	Group B Unsuccessful MI ablation^b^ (n = 5)	Group C PMF with MI pseudo‐block (n = 5)	*P* value
Pre‐procedural characteristics					
Age (y)	62.4 ± 10.1	62.5 ± 10.2	66 ± 12.7	57 ± 5.8	0.3652
Male gender, n (%)	45 (62.5%)	37 (59.7%)	5 (100%)	3 (60%)	0.1995
Left atrial diameter (mm)	43.7 ± 2.7	43.3 ± 2.4	48 ± 2*	44.2 ± 2.9	0.0004
Left ventricular ejection fraction (%)	52 ± 5.5	52.4 ± 4.6	47 ± 13	52 ± 4.5	0.1107
CHA_2_DS_2_‐VASc score	1.86 ± 1.28	1.87 ± 1.27	2.4 ± 1.52	1.2 ± 1.09	0.3327
Previous AF ablation, n (%)	37 (51.4%)	29 (46.8%)	4 (80%)	4 (80%)	0.1492
Clinical arrhythmia at the time of procedure					
AF, n (%)	40 (55.6%)	38 (61.3%)	1 (20%)	1 (20%)	0.0512
AT, n (%)	32 (44.4%)	24 (38.7%)	4 (80%)	4 (80%)	
Procedural characteristics					
Epicardial approach, n (%)	33 (45.8%)	28 (45.2%)	3 (60%)	2 (40%)	0.7851
MI ablation time (min)					
Total	10.73 ± 6.16	10.73 ± 6.14	18.7 ± 9.51**	10.16 ± 2.93	0.0262
Endocardial	10.12 ± 5.87	10.27 ± 5.86	17.66 ± 8.54***	9.96 ± 3.14	0.0313
Epicardial	1 ± 0.54	0.96 ± 0.49	1.73 ± 0.46****	0.5 ± 0.42	0.0005
MI line dimensions					
Length (mm)	32.0 ± 4.3	31.7 ± 4.2	36.2 ± 1.8	32.2 ± 5.0	0.0735
Width (mm)	13.8 ± 4	13.7 ± 3.6	18.6 ± 6.6*****	11.2 ± 3.6	0.0088
Peri‐mitral conduction time Post‐MI block^c^					
Counterclockwise direction (ms)^d^	153 ± 42	154 ± 43	—	146 ± 22	0.6834
Clockwise direction (ms)^e^	157 ± 43	158 ± 44	—	149 ± 21	0.6536
Post‐procedural characteristics and outcome					
Follow‐up period (mo)	22.2 ± 8.1	21.7 ± 8.4	27.2 ± 6.1	24.6 ± 4.9	0.2845
AT/AF recurrence, n (%)	16 (22.2%)	13 (21%)	2 (40%)	1 (20%)	0.6111
AT or mainly^f^ AT recurrence, n (%)	2 (2.8%)	2 (3.2%)	0	0	0.7682
AF or mainly^f^ AF recurrence, n (%)	14 (19.4%)	11 (17.8%)	2 (40%)	1 (20%)	
Post‐procedural antiarrhythmic medication^g^, n (%)	9 (12.5%)	6 (9.7%)	2 (40%)	1 (20%)	0.1246
Amiodarone, n (%)	5 (6.9%)	3 (4.8%)	2 (40%)	0	0.2317
Other, n (%)	4 (5.6%)	3 (4.8%)	0	1 (20%)	

Abbreviations: AF, atrial fibrillation; AT, atrial tachycardia; CS, coronary sinus; MI, mitral isthmus; PMF, perimitral atrial flutter.

^a^ After the first ΜΙ ablation attempt.

^b^ Unsuccessful MI ablation after the first procedure (n = 4) or PMF occurrence due to overt MI conduction recurrence (n = 1).

^c^ The time of impulse rotation around the mitral annulus after achieving MI block according to the conventional pacing maneuvers.

^d^ It is calculated with pacing from a catheter located just anteriorly to the MI line, by measuring the time from the spike to the distal CS bipole.

^e^ It is calculated with pacing from distal CS, by measuring the time from the spike to the catheter located just anteriorly to the MI line.

^f^ More than 50% of the documented arrhythmia burden.

^g^ Systemic AAD therapy, over a period of more than 3 mo within the follow‐up period (excluding the first 3 mo of the blanking period).

* Group B vs Group A, *P* = .0002; Group B vs Group C, *P* = .0396.

** Group B vs Group A, *P* = .0209.

*** Group B vs Group A, *P* = .0246.

**** Group B vs Group A, *P* = .003; Group B vs Group C, *P* = .0004.

***** Group B vs Group A, *P* = .0207; Group B vs Group C, *P* = .0090.

### 3.4 Arrhythmia recurrences and details of the redo procedures

After the index procedure which included MI ablation, a redo procedure was required in nine patients. The AF/AT free survival rate, in a mean follow‐up period of 22.2 ± 8.1 months, was 66.7% (48/72 patients) and 77.8% (56/72 patients) after the index MI ablation procedure and the supplementary redo procedure, respectively. Particularly, in patients with AT, the event‐free survival rate was 71.9% (23/32 patients) and 87.5% (28/32 patients) after the index and the redo procedure, respectively, while patients with AF had 62.5% (25/40 patients) and 70% (28/40 patients) event‐free survival rate after the index and the redo procedure, respectively. Patients with AT appeared to have a significantly better prognosis in maintaining SR after MI ablation than patients with AF (log‐rank, *P* =.0466) (Figure S2). However, 87.5% of patients with AT had previously undergone LA ablation compared to 22.5% of patients with AF. Of the four patients who had unsuccessful MI ablation at the initial procedure, one who relapsed with PMF has undergone re‐ablation with successful completion of MI and since then he is in SR, while of the other three, two had AF recurrence and underwent rate control therapy and one remained in SR. Among patients with PMF and MI pseudo‐block, only one recurred with a brief episode of paroxysmal AF 18 months after the index MI ablation procedure.

After the successful MI ablation, nine patients underwent a redo procedure due to AT/AF recurrence. In 8/9 (88.9%), the pacing proof of MI block was maintained. In one of the nine patients, the MI conduction had overtly recurred, and in two patients, MI pseudo‐block was confirmed, as the pacing criteria of MI block were fulfilled in parallel with the occurrence of PMF. The PMF was successfully treated by ablating the gap in MI.

Thirty‐seven patients who underwent MI ablation had previous AF ablation (33 one procedure and four two procedures, 1.1 procedures/patient). During the MI ablation procedure, PV reconnection was found in 27/37 (73%) patients with at least 1 PV reconnected and in 66/148 (44.6%) of the re‐evaluated PVs.

### 3.5 Complications

In our series, we had only one major complication. A 47‐year‐old male patient, who was ablated for long‐standing persistent AF, 15 days after the procedure, being on rivaroxaban, presented with cardiac tamponade and moderate left pleural effusion. The patient had undergone his first AF ablation in which PVI, box lesion, and successful MI ablation (without CS epicardial access) was performed. The patient was promptly treated with pericardiocentesis, and after a short hospital stay, he fully recovered. To date, he is free of any atrial arrhythmia recurrence for a follow‐up period of 29 months.

## 4 DISCUSSION

### 4.1 Main findings

1. After MI ablation, despite the evidence of complete and bidirectional MI block demonstrated by conventional pacing maneuvers, it is possible for PMF to occur in 6.9% of patients, as shown in our series.
2. The acute success of MI ablation is high, as shown in our study, where 77.8% of patients remained free of arrhythmia at a mean follow‐up period of 22.2 ± 8.1 months, with a significantly better prognosis in the group of patients whose clinical arrhythmia was ΑΤ compared with those presented with AF.
3. The lowest CV in PMFs with MI pseudo‐block is significantly lower than the lowest CV in usual PMF circuits and also in PMF circuits of patients who have previously undergone additional ablation in the anterior LA.

### 4.2 Interventional outcome

In our series, we had a satisfactory acute success rate (~96%) in achieving complete MI block. Probably, our systematic approach with the steerable sheath support, the continuous maintenance of sufficient catheter contact force (>15 g), and the accuracy of the 3D imaging—there were no patient movements due to general anesthesia—contributed to this high percentage.^23^ However, as shown by other studies,^24^ there will always be a considerable group of patients in whom we cannot achieve complete MI block with the available technological means. Thus, in our study, if we add the percentage of interventional failure (4.2%) and the percentage of patients presenting with PMF and MI pseudo‐block (6.9%), we reach an 11.1%. These patients have a higher likelihood to develop PMF even if this arrhythmia is not pre‐existing, a fact that we need to take into consideration when we intend to perform the MI line.^14^, ^15^, ^16^ Presumably, reinduction attempts should be performed only to exclude PMF induction after each successful MI ablation. On the contrary, the reinduction of AF, despite some relatively contradictory data,^25^ does not seem to be a predictor in maintaining SR.^26^ In our study, we also found that MI ablation is a time‐consuming procedure with the mean pure ablation time being almost 11 minutes, something that we must always take into account for the preparation of the procedural plan.

### 4.3 Technical aspects of MI ablation

**FIGURE 6:**
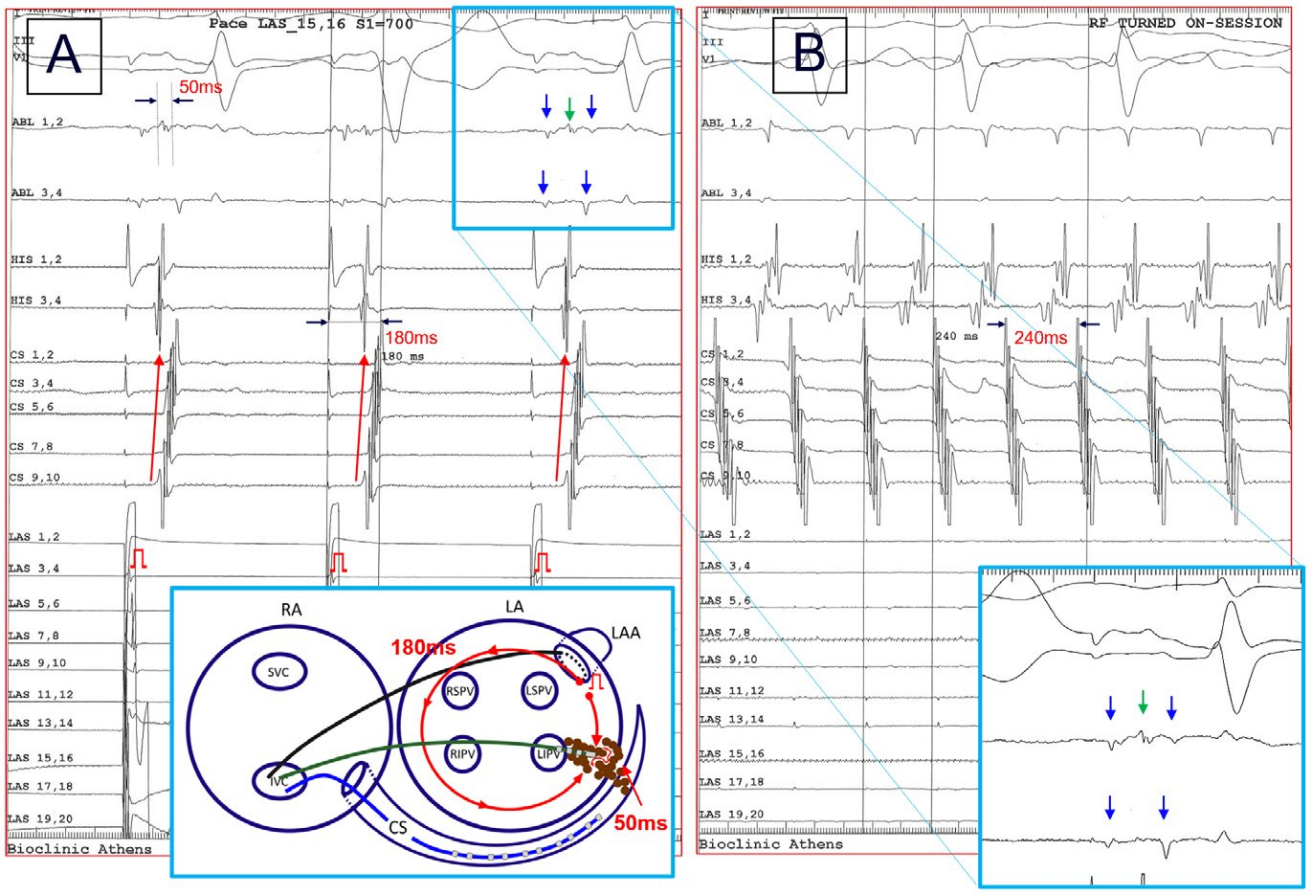
(A) Pacing from the left atrial appendage (LAA) showing the reversal of the activation sequence in the coronary sinus (CS). The time of the counterclockwise rotation from the spike to the distal CS was 180 ms The ablation catheter is located in the mitral isthmus (MI) line and records double far‐field potentials indicative of conduction block (blue arrows) and in between a high frequency near‐field electrogram (green arrow) with total duration 50 ms The latter is possibly indicative of residual conduction through the MI ablation area; it does not have enough time to cross the MI and eventually fades colliding with the counterclockwise wavefront. (B) Clockwise perimitral atrial flutter with cycle length 240 ms that was diagnosed with high density activation mapping using the 3D mapping system

In the process of MI ablation, the most prominent demonstration of successful completion of the linear lesion is the clear reversal of the activation sequence in the CS during pacing from the LAA. For the MI block confirmation, we used pacing criteria indicative of bidirectional change in the activation sequence of the peri‐mitral region and not simply the increase in MI conduction time or the recording of double potentials which are more uncertain criteria.^27^ It seems that, in the same manner as in CTI ablation, the activation sequence shifts abruptly^28^ In about half of the cases, epicardial access was required in our study; the sequence in CS was reversed with minimal energy delivery within the CS. The extent of the lesions delivered epicardially through the CS does not anatomically extend to the entire epicardial aspect, obviously because there is no free access to a much wider area, but only to the muscular bundles surrounding the CS. This may create a selective block towards the CS multipolar catheter but not to the entire MI. Presumably, some cardiac bundles may remain intact, and as we have learned, these may be located in the myocardial coverage of the vein of Marshall, the complex anatomical region of the LA ridge, the lateral aspect of the LAA base, or the area between the LAA and the mitral annulus.^18^, ^19^, ^20^, ^21^ Apparently, the impulse is passing through the remaining MI connections with a very long delay. Due to this fact we have an apparent change in activation sequence as the impulse from pacing beside the line is delayed in its passage through the MI lesions going much faster in the opposite direction (Figure 6).

In the study by Barragan et al,^18^ it was shown that the breakthroughs are in the majority (8/11) epicardial. In our study all the PMFs with MI pseudo‐block were ablated from the endocardial site. This can be explained in three ways: (a) the conduction gap is located in the endocardial site; (b) more endocardial ablation is needed to create a transmural lesion; or (c) we finally ablate the endocardial breakthroughs of epicardial connections,^19^ although the critical lesions, in our five cases, were located in juxtaposition with the MI line. In the study of Fujisawa et al,^20^ the pseudo‐block was observed in 33.3% of the cases. In this study, the MI ablation was attempted at a higher level in contrast to our series where the initial choice was the 4 o'clock site of the mitral annulus (LAO view), possibly lower than the position of the ligament of Marshall and posteriorly of the lateral border of the LAA. If the MI line cannot be performed, alternatively other linear lesions were attempted to interrupt MI conduction or PMF circuits,^29^, ^30^ something that we attempted in our study in case of failure to achieve complete MI block. Probably, one way to avoid residual ΜΙ connections, beyond the re‐induction challenge mentioned above, is the detailed mapping in the boundary of the line and the identification of near field potentials representing conduction gaps.

### 4.4 Conduction velocities and implications for PMF ablation

In our study, we showed that PMF in patients with MI pseudo‐block is an arrhythmia with special electrophysiological features. The evaluation of the activation maps revealed a very slow conduction through a narrow excitable channel in the area of the previous lesions. On the contrary, measuring the CV with high‐density mapping in a large number of PMF that occur after AF ablation, we found that the CV does not show large differences throughout the course of the circuit. This also applies to patients with PMF who have had previous ablation in LA beyond PVI. In addition, the presence of conduction channels was found in a very small percentage of PMF circuits. Therefore, it appears that in the majority of PMFs after AF ablation, there are areas where the CV is slower, but usually there is no area with marked conduction delay. This reasonably raises the question of what the most secure strategy for PMF ablation could be to ensure no recurrence. If we target the area of the lowest CV or the narrowest corridor, we can temporarily stop the circuit, but there may still be pathways throughout the extent of the perimitral area that may allow its re‐initiation. Nevertheless, it is doubtful whether the area around a channel in a propagation map is truly unexcitable or could become excitable in a different waveform direction. Thus, it seems reasonable that the most secure way to prevent PMF is to interrupt the circuit in the MI. Perhaps a useful example is the ablation of CTI‐dependent flutter, where the arrhythmia can be terminated even with the onset of ablation, if a critical area is affected, but there is no doubt that the circuit can relapse unless complete and bidirectional CTI block is eventually achieved.

In several recent series with high‐density mapping, targeting the functional rather than the anatomical isthmus has been the main ablation strategy.^11^, ^12^, ^13^ It should be considered that many of these studies^12^, ^13^ have been performed with the Rhythmia system, which actually creates high density maps but probably does not facilitate the linear ablation with complete and transmural lesions, as this system did not have catheters with contact force technology. According to our previous observation in a longitudinal patient cohort with ATs after AF ablation, targeting the macro‐reentrant circuits of the LA with ablation of the anatomical isthmuses has better results in maintaining SR.^31^ However, we must recognize that all these views are based on empirical observations. Besides, the question of whether we should ablate the anatomical or the functional isthmus is difficult to answer even by a randomized study, as each case has specific and unique features and so it is difficult to implement a preplanned ablation strategy.

### 4.5 Long‐term outcome

Our study showed that patients with AT had a better prognosis in maintaining SR than those with AF. Evidently, it should not be overlooked that patients with AT had a much higher rate of previous LA ablation than patients with AF. Apparently, during the AT ablation, apart from the MI, additional ablation was carried out to treat other ATs or to re‐isolate the PVs. Probably, when ΑΤ becomes a clinical arrhythmia after AF ablation, a less complex substrate is created, which if mapped in detail, can be ablated with great success and favorable long‐term results.^32^

In the redo cases, we found the pacing‐proven maintenance of MI line at a rate of 86%. On the other hand, the maintenance rate of PVI per patient with at least one PV reconnected and per PV was 27% and 55%, respectively, after 1.1 previous AF ablation procedures. Our findings are consistent with the study by Mujiovic et al^33^ in which the invasive re‐evaluation, 3 months after the index procedure, showed numerically higher maintenance rate in linear lesions than in PVI. We do not know why this happens. It is possible that the greater persistence on a limited surface and the definite procedural endpoint in MI ablation lead to more transmural lesions.

### 4.6 Limitations

In our study, we found a significant proportion of patients with PMF after conventional proof of complete block. However, we cannot ignore the fact that there may be cases with pseudo‐block without subsequent PMF recurrence, as the re‐induction challenge was performed only after AT ablation and not systematically in the context of AF ablation.

Regarding patients who developed PMF after confirmation of MI block in the same procedure, the recurrence of MI conduction in an “on–off” manner probably cannot be excluded, although the pacing confirmation was always performed very close to PMF occurrence. On the other hand, patients who developed PMF in another procedure and were found from the beginning to meet the pacing criteria for MI block should confidently be considered to have MI pseudo‐block.

Another limitation is that the CV measurement is governed by subjective selection of the acquired points. By taking different points for this measurement or even by shifting the timing of the isochronal scale, it can lead to significant differences in the CV value. However, all these measurements were made in high‐density activation maps, a fact that mitigates the subjectivity in CV evaluation. In addition, the significant difference found between PMFs with ΜΙ pseudo‐block and usual PMFs that occur after AF ablation probably circumvents the above limitation.

## 5 CONCLUSIONS

Mitral isthmus ablation is a process that, if performed diligently, achieves a high rate of acute success. In a small percentage of patients, probably due to the anatomical complexity of MI, incomplete ablation lines could permit the occurrence of PMF circuits despite the verisimilar proof of MI block. These tachycardias are reentrant circuits characterized by a large deceleration of CV in the area of previous lesions in the MI. In this particular feature, they differ from ordinary PMF circuits, in which the CV does not show huge differences in the whole extent of their course. This fact is probably advocating for ablation directed at anatomical rather than functional isthmuses.

Moreover, in our study, we found that patients who underwent ΜΙ ablation and had ΑΤ, usually after previous AF ablation, had a much better prognosis than those who underwent MI ablation presenting with AF. Probably, after a sufficient substrate modification the less complex electrophysiological environment can only maintain ATs, which if mapped in detail and treated in a tailored manner, are very likely not to recur.

## CONFLICT OF INTEREST

The authors have no conflicts of interest relevant to this manuscript.

## Supporting information

Video S1

Video S2

Video S3

Video S4

Video S5

Video S6

Video S7

Supplementary Material
