# Supplementary material for: Incidence, electrophysiological characteristics, and long‐term follow‐up of perimitral atrial flutter in patients with previously confirmed mitral isthmus block
Source: J Arrhythm. 2021 May 12;37(3):584–96. doi: 10.1002/joa3.12545 (PMC8207388; doi:10.1002/joa3.12545)
Supplement: Supplementary file 8 — Supplementary Material [file JOA3-37-584-s003.docx]

Manuscript Title:

Incidence, electrophysiological characteristics, and long-term follow-up of perimitral atrial flutter in patients with previously confirmed mitral isthmus block

Panagiotis Ioannidis, et al.

**Supplementary Figures**


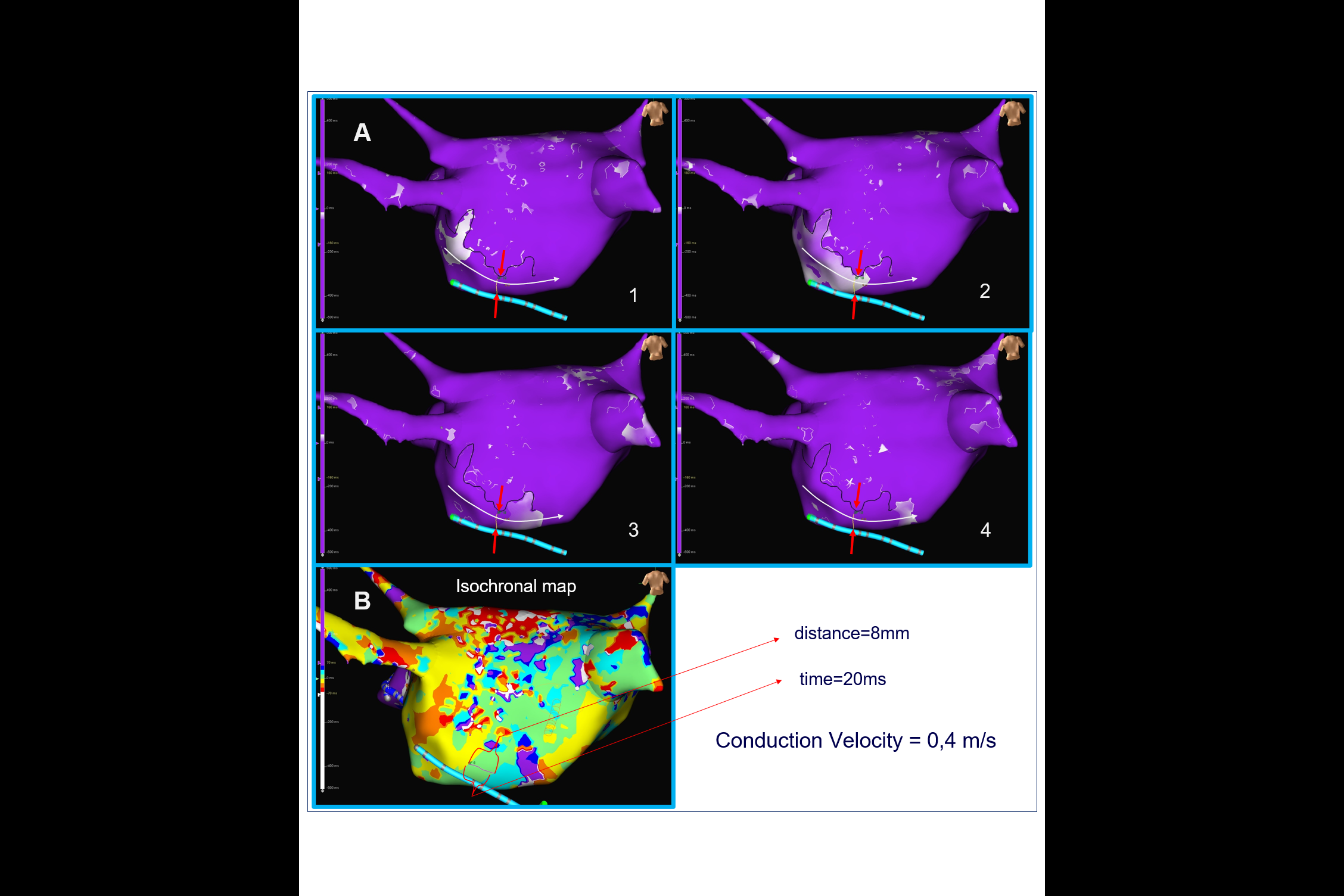


**Figure S1.** A: Propagation map of a clockwise PMF with a narrow conduction channel (red arrows) in the lower mitral annulus (video 7). B: The CV in this area was manually measured 0.4 m/s.


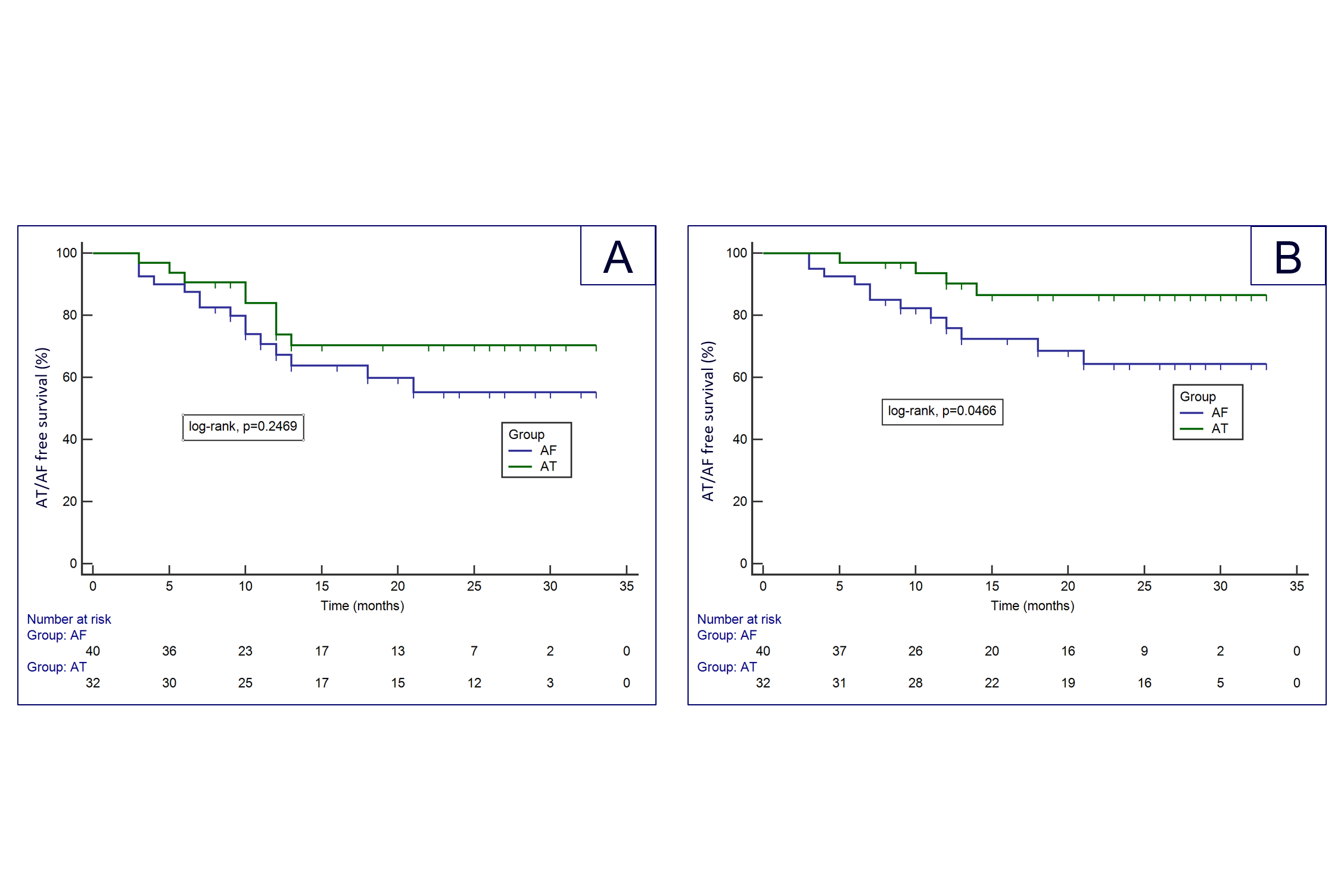


**Figure S2.** Kaplan-Meier curve of AT/AF free survival in patients with AT or AF after the index MI ablation (A) and the subsequent redo procedures (B).

Video 1. An impulse propagation video of a clockwise PMF in a patient with MI pseudo-block. The detailed mapping of the posterior MI showed the breakthrough point with very slow conduction velocity.

Video 2. Septal and lateral view of the LA showing the impulse propagation of a clockwise PMF in a patient with MI pseudo-block. The impulse is slowly propagated through a narrow corridor (between the red lines) perpendicular to the previous MI ablation line.

Video 3. The ablation at the breakthrough point of the PMF described in Video 2 restores the SR. A sufficient pressure of 10-30 g had to be exerted during ablation.

Video 4. The detailed propagation mapping shows the breakthrough point of a clockwise PMF in a patient with MI pseudo-block.

Video 5. The propagation video of a clockwise PMF in a patient with previous PVI and complex fractionated atrial electrograms ablation (gray areas in the anterior LA). Notice that the conduction velocity of the wavefront does not show significant differences throughout the circuit.

Video 6. A typical counterclockwise PMF in a patient with a previous PVI ablation.

Video 7. A posterior view of the LA in a patient with clockwise PMF and previous PVI and complex fractionated atrial electrograms ablation showing a narrow conduction channel with a width of 10 mm in the lower mitral annulus. The CV in this area was manually measured 0.4 m/s (see Figure S1).
